# Case report: Carnivore–ketogenic diet for the treatment of inflammatory bowel disease: a case series of 10 patients

**DOI:** 10.3389/fnut.2024.1467475

**Published:** 2024-09-02

**Authors:** Nicholas G. Norwitz, Adrian Soto-Mota

**Affiliations:** ^1^Harvard Medical School, Boston, MA, United States; ^2^Metabolic Diseases Research Unit, National Institute for Medical Sciences and Nutrition Salvador Zubiran, Mexico City, Mexico; ^3^Tecnologico de Monterrey, School of Medicine, Mexico City, Mexico

**Keywords:** carnivore diet, Crohn’s disease, inflammatory bowel disease, ketogenic diet, ulcerative colitis

## Abstract

**Background:**

Very-low-carbohydrate diets, including ketogenic and carnivore diets, are gaining popularity for the experimental treatment of a wide range of disorders, including inflammatory bowel disease (IBD).

**Methods:**

Participants were recruited through a social media survey. Final inclusion required a histologically confirmed diagnosis of ulcerative colitis (UC) or Crohn’s disease that was responsive to treatment with a ketogenic or carnivore diet without medication or with successful medication cessation on the diet. Clinical improvement was measured with the Inflammatory Bowel Disease Questionnaire (IBDQ).

**Results:**

We report on 10 cases of IBD responsive to ketogenic, mostly carnivore, diets. Clinical presentations were diverse, including six cases of UC and four of Crohn’s disease. Clinical improvements were universal, with clinical improvement scores ranging between 72 and 165 points on the IBDQ. Patients’ diets comprised mostly meat, eggs, and animal fats. Patients report their diets are pleasurable, sustainable, and unequivocally enhance their quality of life.

**Conclusion:**

Ketogenic and carnivore diets hold promise for the treatment of IBD, including UC and Crohn’s disease. These cases are consistent with clinical literature that shows an inverse association between intestinal ketone levels and IBD activity, as well as the therapeutic effects of low residue elimination diets on colonic microbiota metabolism.

## Graphical abstract

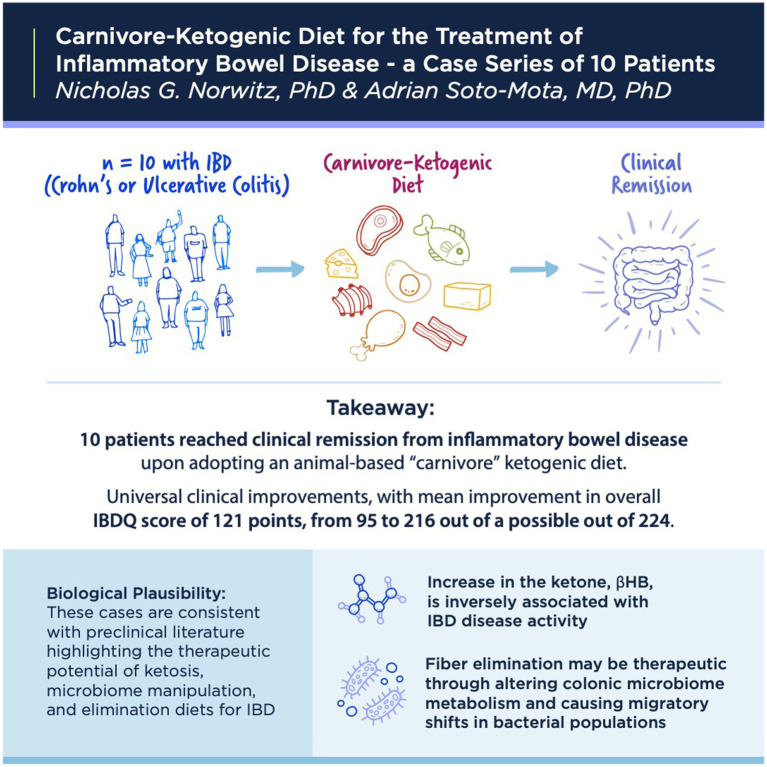

## Introduction

Very-low-carbohydrate ketogenic diets (KDs), long-known and well-studied as a therapeutic option for pediatric epilepsy (1), are becoming increasingly popular for the clinical treatment of a broad range of medical conditions. Public awareness of KDs mostly focuses on applications related to obesity and diabetes, but there is also accumulating data for KD in other conditions. This includes preclinical and clinical interventional data for certain mental health disorders (2, 3), chronic infectious disease (4), and human randomized controlled trial data for Alzheimer’s disease (5, 6) and polycystic kidney disease (5), to name a few examples.

The therapeutic potential of KD may derive from a combination of three features of the diet:

1. Carbohydrate reduction can facilitate weight loss and improve glycemic control in those with obesity, metabolic syndrome, pre-diabetes, or diabetes (7–9).
2. The restrictive nature of the diet often facilitates the elimination of “problem” foods, including those that may act as immune triggers.
3. Ketone bodies generated by the liver are potent regulatory and signaling molecules, akin to hormones as much as they are fuel substrates. They regulate the immune system and metabolism through cell surface receptors, inflammasome inhibition (10), histone deacetylase (HDAC) inhibition (11), epigenetic regulation, and by acting as post-translational modifiers through the process of lysine β-hydroxybutyrylation on >1,000 different proteins (12).

Another, often overlapping, form of a very low-carbohydrate diet is the “carnivore diet,” in which subjects eat almost exclusively meat, seafood, eggs, animal fats, and dairy products. Although minimally studied, a recent cohort study from Harvard University of 2,029 subjects consuming a carnivore diet for ≥6 months reported, “*Contrary to common expectations, adults consuming a carnivore diet experienced few adverse effects and instead reported health benefits and high satisfaction*” (13). There is also a rapidly growing community on social media reporting improvements in health status for a broad range of conditions following the adoption of experimental carnivore diets. The rising popularity of the diet demands further study for its safe and targeted use.

The therapeutic potential of human carnivory has biological plausibility, given it is by nature an elimination diet and usually induces a state of ketosis. In this case series, we report on 10 patients who successfully treated IBD with a KD or carnivore diet. We report on their experiences, discuss potential biological mechanisms for KD and carnivore in IBD, highlight concerns about the diet, particularly with respect to its impact on lipids, and make suggestions for the future direction of research.

## Methods

### Recruitment

Participants were recruited from authors’ prior awareness of patient cases and/or response to an announcement on social media, distributed via Twitter. Potentially interested participants were interviewed by one of the authors and asked to provide medical documentation to support key elements in their histories, including confirmation of UC or Crohn’s disease from colonoscopy/endoscopy. The final 10 patients were selected based on the order of response to solicitations, provided they could produce evidence of a histological diagnosis, i.e. cases were determined *a priori*, not after interviews. No cases of patients with IBD responding that a KD or carnivore diet exacerbated their condition were received.

**Table 1:** Treatment of inflammatory bowel disease with ketogenic and carnivore diets.

Patient	Inflammatory bowel disease	Age at diagnosis	Prior diet(s)	Duration of remission on carnivore-ketogenic diet
1 (TH)	Crohn’s	**30**	Red meat-free, Gluten-free	**5 years**
2 (AN)	Ulcerative colitis	**25**	SAD, Paleo	**11 years**
3 (OS)	Crohn’s	**20**	SCD	**5 months**
4 (IA)	Ulcerative colitis	**30**	SAD, Gluten-free, Whole 30	**6 years**
5 (MI)	Ulcerative colitis	**18**	SAD, Sugar-free	**3 years**
6 (NE)	Crohn’s	**12**	Vegan, Vegetarian	**6 months**
7 (VI)	Crohn’s	**14**	SAD, Paleo	**>1 year**
8 (TA)	Ulcerative colitis	**38**	Vegan	**>1 year**
9 (BL)	Ulcerative colitis	**21**	SCD, Low FODMAP, Vegetarian, Vegan	**4.5 years**
10 (E)	Ulcerative colitis	**41**	SAD, Calorie-restriction	**>1 year**

Patient diagnoses, age at diagnosis, prior diets, current diet, and duration of remission on current diet are provided. Duration of remission is considered the contiguous time, to date, during which the disease is in clinical remission. SAD, standard American diet; SCD; specific carbohydrate diet. No patients were on concurrent medications for IBD at the time of the interview.

### Ethics

Written, informed consent was obtained from the participants for the publication of this case series and any potentially identifying information/images.

All patients were shown a written version of the section of their report and asked to confirm details prior to consent.

Patients are presented with pseudo-initials to protect their identities. IRB approval was provided by the Medica Sur Human Research Ethics Committee with the code 2024-RC-011 in July 2024.

### Supporting data

All included patients were required to provide evidence of histologically confirmed IBD via medical records and were also asked to provide available past and present laboratory results.

Patients were additionally asked to keep prospective 48 h food logs with as much detail as possible, including mass intakes, fat percentages, specific food brands, and photographic documentation for author review. Dietary data were extracted for these records and are presented in Supplementary Table S1.

All patients filled out IBDQ-32 questionaries to evaluate their clinical condition before and on a carnivore-KD.

## Case descriptions

A summary of patient case histories is presented in Table 1, and IBDQ-32 results are presented in Table 2.

**Table 2:** Inflammatory bowel disease quality of life (IBDQ) 32-question results.

Patient	Bowel		Systemic		Emotional		Social		Total (32–224)		
	*Before*	*Present*	*Before*	*Present*	*Before*	*Present*	*Before*	*Present*	*Before*	*Present*	*Change*
1 (TH)	27	69	16	31	47	72	26	35	116	207	**91**
2 (AN)	25	68	21	32	29	79	18	35	93	214	**121**
3 (OS)	33	66	11	33	37	74	15	34	96	207	**111**
4 (IA)	26	70	5	35	24	84	28	28	83	217	**134**
5 (MI)	51	69	24	35	53	84	23	35	151	223	**72**
6 (NE)	42	70	22	33	53	80	25	35	142	218	**76**
7 (VI)	18	69	10	35	18	80	7	34	53	218	**165**
8 (TA)	16	68	7	33	36	84	15	35	74	220	**146**
9 (BL)	19	64	6	34	31	77	14	35	70	210	**140**
10 (E)	18	70	10	34	26	84	13	35	67	223	**156**
MEAN									95	216	**121**

The results are given before the carnivore/ketogenic diet, and while on the diet. Each of the 32 questions is rated 1 to 7, with 1 being the “worst” and 7 being the “best.” Questions are grouped into 4 categories with (min – max scores) as follows: bowel (7–70), systemic (5–35), emotional (12–84), and social (5–35), for a total score (32–224).

### Case 1

Patient TH is a 62-year-old man who was diagnosed with Crohn’s at age 30 and has no other significant medical history. TH first started to develop symptoms at age 28, including gas, bloating, and urgency with loose stools. At the time, he was eating a standard American diet. At the age of 30, as a second-year student in law school, he was diagnosed with Crohn’s disease. He was treated with prednisone for flares and oral sulfasalazine, with minimal response. He discontinued the sulfasalazine at age 35. He transitioned to oral mesalamine, also without any noticeable therapeutic benefit. At times, eating was so uncomfortable that he consumed only fluids, and lost 80 lbs. He underwent a terminal ileectomy at age 35. Between the ages of 23 and 48 years, he avoided red meat for perceived health benefits and environmental concerns, finally breaking his “quarter century beef and lamb fast” after learning about regenerative agriculture. Between the ages of 30 and 50 years, he had persistent symptoms, severe intermittent flares, and more than 10 endoscopies/colonoscopies. At age 50, TH tried a gluten-free diet, with some improvement, but symptoms persisted. Motivated by a change in symptoms, TH started reading more about diet and nutrition and tried a KD at age 56 years, experiencing “90%” perceived symptom improvement. In the first year, his KD was <20 g/d carbohydrate and included diverse “keto” foods, including almond flour and zero-calorie sweeteners. His BMI decreased from 29.5 kg/m^2^ to 24.0 kg/m^2^. However, colonoscopy/endoscopy still showed some Crohn’s activity in the neoterminal small intestine, albeit with resolution in the large intestine, and his doctor recommended immunomodulatory biologics to reduce the risk of future surgeries. He discontinued mesalamine and underwent regimens of adalimumab and then ustekinumab each for approximately 6 months, with no further perceived benefit from either. At age 57, he tried a fully carnivorous diet, including mostly beef, and eggs, with minimal dairy, and black coffee. At age 58, he began adding back small amounts of cooked cruciferous vegetables and fermented foods. Most recent labs on a carnivore diet include CRP 0.6 mg/L, HbA1c 5%, fasting insulin 3.1 uIU/ml, LDL 65 mg/dL (10 mg/dL lower than prior to carbohydrate restriction), HDL 60 mg/dL, and triglycerides 53 mg/dL. Since transitioning to a whole food, mostly carnivore KD, urgency and pain have completely resolved, with mostly formed stools, “99%” overall improvement in symptoms, and no signs of Crohn’s activity in the small or large intestines on the latest colonoscopy/endoscopy at age 59.

#### Patient perspective

A keto-carnivore diet let me forget that I have Crohn’s. No drugs, no surgery… The decades that I dealt with Crohn’s are like another lifetime.

### Case 2

Patient AN is a 52-year-old man diagnosed with ulcerative proctosigmoiditis at age 25 with no other significant medical or family history. His initial symptoms included loose and bloody stools 5–8 times daily that persisted for several months before he presented to his primary care provider, who referred him for a colonoscopy, upon which his UC was diagnosed. He was treated with a hydrocortisone enema followed by oral mesalamine, which he continued to take for the next 5 years. Over this time, he continued to struggle with occasional loose and bloody stools and general gastrointestinal distress. His diet at the time was consistent with a standard American diet, including pizza, diet sodas, and a generally mixed macronutrient intake. At age 30, after reading about low-carbohydrate diets, he changed his diet to one focusing on low-carb whole foods. He eliminated diet sodas and, on a given day, would consume mostly animal protein (meat, chicken, and eggs), nuts (almonds and almond butter), and low-carb vegetables (broccoli) with some fruit. Over the next 12 years, he progressively reduced his carbohydrate intake, slowly transitioning to a KD. With his change in diet, AN experienced an improvement in symptoms, with the elimination of loose and bloody stools. He gradually reduced his oral mesalamine dose and eventually came off the medication completely, remaining in remission with minimal symptoms. Over this time, he had four colonoscopies, the last at age 42, showing no signs of disease activity, consistent with parallel improvements in his symptoms with carbohydrate reduction. His current carnivore-KD consists of mostly red meat, eggs, and small amounts of low-carbohydrate plant matter (mushrooms, avocado, and macadamia nuts), with no symptoms. With his dietary changes, AN also reduced his BMI from 25.8 kg/m^2^ to 23.3 kg/m^2^, and he remains physically active. He reports feeling “the best I’ve ever felt.” Lab markers are significant for fasting insulin <3 uIU/ml, CRP <0.5 mg/dL, AST 24 U/L, and ALT 27 U/L. Lipids upon last check on a carnivore diet were total cholesterol 479 mg/dL, LDL-C 399 mg/dL, HDL-C 70 mg/dL, and triglycerides 83 mg/dL, a lipid panel trending toward the lean mass hyper-responder (LMHR) phenotype (14–17). He refused pharmacotherapy for hyperlipidemia. CAC score performed at age 51 included LM 0, LAD 2.54 (27^th^ percentile), LCx 0, RCA/PDA 0, and “minimal [total] plaque burden.”

#### Patient perspective

I’m healthy. I cured myself of colitis with lifestyle when medications were not doing enough. My doctor clearly does not seem fully informed with what’s going on in my body and with my lipids, so I do not feel comfortable taking any medications unless he can convince me why I should take them.

### Case 3

Patient OS is a 31-year-old woman with a family history strongly significant for IBD in 1 sister, 1 brother (of 7 total siblings), and 10 first cousins, who was herself diagnosed with Crohn’s disease at age 20 during the first trimester of her first pregnancy. Her initial presenting symptoms included severe mouth sores that made it difficult to eat. She would place food in her mouth and let it dissolve, rather than chew. Over her first trimester, she lost weight, dropping from a BMI of 21.0 kg/m^2^ to 16.5 kg/m^2^, finally achieving a BMI of 21.9 kg/m^2^ by delivery at 36 weeks. She was diagnosed with Crohn’s disease by colonoscopy during the pregnancy. Because of the severity of her condition, she was treated with certolizumab, an anti-TNFɑ, but without a significant improvement in symptoms. Mercaptopurine and a specific carbohydrate diet were added to her regimen, although she discontinued the former after a year. For the next 10 years, on certolizumab and a specific carbohydrate diet, she continued to experience flares with persistent loose stool with occasional blood, chronic anemia, chronic yeast infections, and fatigue. In June 2022, her medication was changed to adalimumab, but without a response as assessed by continued symptoms, sigmoidoscopy, and a persistently elevated calprotectin. Her doctor then recommended a trial of risankizumab; however, OS expressed interest in trialing a carnivore diet, first based on the positive experiences of a non-blood relative who had success with a carnivore diet for Crohn’s and who reports 5 years of disease remission off medications (this relative is not included in this case series). OS’s physician agreed to support her and she adopted a carnivore diet. Over the first 5 months on the carnivore diet, her calprotectin dropped from 3,300 to 870 μg/g. To date, she reports no symptoms at all while on the diet, and no loose stools except on the few occasions when she diverged from a carnivore diet. She has discontinued all her medications with her doctor’s awareness and supervision.

#### Patient perspective

Within days my bowel movement changed from loose to normal! And within 2 weeks, I had more energy than I did the past 10 years. When I last spoke with my doctor he said that if I’m feeling incredible he does not want to put me on the medication. And that was so encouraging! Especially since he has been really skeptical of the diet.

### Case 4

Patient IA is a 40-year-old man with a significant medical history of brain surgery in 2006 for arteriovenous malformation who was diagnosed with UC at age 30. IA began having symptoms of gastrointestinal distress starting in his teenage years, including loose stools, bloating, and urgency, with progression in symptom severity over the years. By the time of diagnosis, he was having 15 loose stools per day. A colonoscopy revealed disease in the descending and sigmoid colon. At the time of diagnosis, he was consuming a standard American diet. He was initially prescribed oral mesalamine but instead wanted to trial dietary therapy. He first tried a gluten-free diet for 5 months, followed by a paleo diet for 10 months, and subsequently various elimination whole food-based diets inclusive of whole vegetables and fruits, but with persistence of symptoms. At the time, his BMI was 30.3 kg/m^2^. Approximately at age 34, he committed himself to a “meat-only” diet, including beef, chicken, pork, and fish, based on his hope that eliminating fermentable plant matter might provide some symptom relief. IA reports that within 4–5 days “all my symptoms subsided,” and he went from having 8–10 loose stools per day to one formed stool in the morning. After a month, he added back eggs and raw dairy, with continued absence of IBD symptoms. Over time, he shifted his diet to one emphasizing “99% beef and eggs, with sashimi once per month,” cooking his meat and eggs exclusively in raw butter and beef tallow. His BMI dropped to 25 kg/m^2^, and a recent lipid panel was consistent with an LMHR phenotype (14–17), with total cholesterol 392 mg/dL, LDL-C 268 mg/dL, HDL-C 91 mg/dL, triglycerides 51 mg/dL, hsCRP 0.6 mg/L, and a CAC score of 0 at age 39 years. He said he would consider statin therapy if functional testing (CCTA/CAC) shows any evidence of disease but is not presently on pharmacotherapy. When, over the past 6 years, IA has tried to add back vegetables, honey, or fruit, symptoms gradually return. Otherwise, he has remained in complete clinical remission for the past 6 years.

#### Patient perspective

On the carnivore diet, I feel better than I did when I was a 20-year-old college football player. I’m physically the best I’ve ever been at a lean 170 lbs and can bench 10 reps of 225 lbs. My mental clarity is freakin’ awesome! With the lifestyle, I have not had to worry about my colitis for 6 years.

### Case 5

Patient MI is a 30-year-old man diagnosed with UC at age 18. Months before his diagnosis, he developed loose stools ~6 times per day with occasional blood and tenesmus. He was initially managed with a daily mesalamine suppository, which reduced blood loss, although he continued to have life-impairing frequent loose bowel movements with tenesmus. He reports being consistently on the lookout for toilets because of urgency and that his symptoms created a social obstacle, particularly with potential romantic relationships. At the time, his diet consisted of a standard mixed macronutrient diet with robust intake as a recreational powerlifter. At age 22, BMI was 34.3 kg/m^2^. At age 23, MI started systematically changing his diet with the hope of therapeutic effect. MI found reducing sugar, with a gradual progression to a KD consisting mostly of meat and green vegetables by age 25, significantly improved his symptoms, with only two loose stools per day and moderate bloating. At age 26, he trialed a full carnivore-KD after hearing a medical doctor discuss the experimental treatment. He was astonished to find that, after cutting out the remaining plant material from his diet, his residual symptoms disappeared. He discontinued mesalamine without issue. On a KD, and then carnivore diet, he lost ~30 kg, with a present BMI of 25.1 kg/m^2^, despite reporting very little decrement in his lifts. His current diet is one meal a day, consisting of 1 kg 80/20 beef 500 g flank steak, and 10 large eggs, cooked in 2 Tbsp tallow.

#### Patient perspective

I cured my ulcerative colitis with a ketogenic carnivore diet. I want to share my story in case it can help people. I have friends with UC who know how well I’m doing and, honestly, I do not understand why they do not give it a try. What is there to lose?

### Case 6

Patient NE is a 34-year-old man who was diagnosed with Crohn’s at age 12. At the time of diagnosis, he had been suffering for several months with chronic belly pain and frequent loose stools. He was first treated with infliximab for 2 years, with minimal response, and corticosteroids during flares. At age 15, he transitioned to adalimumab, which he remained on until age 26, experiencing ~1–3 flares per year. At age 26, he trialed vedolizumab, and at age 27, he tried ustekinumab, but neither was able to keep his disease in remission for more than several months. At age 30, after watching a popular documentary promoting veganism, NE tried a vegan diet, which he remained on for 2 years, despite intermittent flares and terrible bloating. At the time, he was aware of the carnivore diet but was extremely skeptical given what “I thought I knew” about nutrition. Gradually, he transitioned to a whole-food vegetarian diet and then an omnivorous diet. After suffering from Crohn’s disease for 22 years, never with lasting symptom relief, he decided to try a carnivore diet because, in his words, “[expletive here] it, I’ll try it for a month. I have nothing to lose.” Within 2 weeks, NE reports having formed stools and reduced bloating. He discontinued ustekinumab, perceiving no benefits from the medication, and for the first time in decades, he remains in remission and free of diarrhea and bloating. With dietary changes, his CRP decreased from 3.1 mg/dL to 0.1 mg/dL.

#### Patient perspective

Carnivore seems limiting at first, but it’s actually freeing.

### Case 7

Patient VI is a 31-year-old man diagnosed with Crohn’s disease at age 14. He first presented with progressively worsening loose stools, sometimes upwards of 10 times daily, with occasional blood. VI’s flares were treated with corticosteroids, and he was put on methotrexate until age 15, then infliximab until age 18, and then azathioprine until age 20, with mild symptom improvement but continuing flares. Approximately at age 20, VI attempted a paleo diet and discontinued azathioprine, citing some symptom improvement with the diet and that he was concerned about the iatrogenic effect. After discontinuation of azathioprine, his symptoms worsened. He was then treated with adalimumab until age 25, followed by ustekinumab and vedolizumab, but his disease remained refractory to treatment. At age 27, he underwent subtotal colectomy with ileostomy for a disease located in the large intestines. At age 29, Crohn’s activity was noted in the terminal ileum. VI felt “devastated.” He had heard about the carnivore diet through discussions on social media and decided, before trying yet another medication, that he wanted to try the carnivore diet. For 4 months, he ate exclusively meat, eggs, bacon, and salt with near-complete resolution, feeling “100% from a GI standpoint.” Since that time, he has remained a carnivore and is in remission. He has liberated his diet slightly, with small volumes of cruciferous vegetables, the inclusion of low-carbohydrate dairy (cheese, Greek yogurt), and an occasional handful of blueberries, but his diet remains >90% meat and eggs. Since starting a carnivore diet, calprotectin had dropped from 4,291 μg/g to 9 μg/g. He is on no medications.

#### Patient perspective

The carnivore diet requires me to restrict what I eat but it’s given me health that I never thought I could have. Worth it. I cannot believe that we are still telling patients that their diet does not really matter with respect to IBD. It’s absolutely bonkers!

### Case 8

Patient TA is a 43-year-old man who was diagnosed with UC at age 38, following 2 years of lethargy, frequent bloody bowel movements, vomiting, and stomach cramps starting at age 36. Following symptom onset, but prior to diagnosis, he adopted a vegan diet; however, this failed to improve his condition. He subsequently developed anemia that required a blood transfusion and abandoned his vegan diet for health and safety reasons. Following his diagnosis of UC by colonoscopy at age 38, he was treated with prednisone and mesalamine. However, this treatment did not improve his symptoms and he started adalimumab with only modest improvement in symptoms. On follow-up, his nutritionist advised a low-fiber diet as an experiment to see if this might improve his symptoms. With symptom improvement, he went on to additionally eliminate other foods and gradually progressed to a full carnivore-KD, largely consisting of red meat, salt, and water since then. Since adopting a carnivore diet, he has been completely free of IBD symptoms, has achieved normal iron studies, and discontinued mesalamine or adalimumab 18 months ago without any change in clinical signs or symptoms.

#### Patient perspective

I feel better than at any point in my life, work out 6 days a week, require no medication, and experience more energy on a day-to-day basis than at any point in my life that I can remember. My health transformation has truly been a miracle.

### Case 9

Patient BL is a 28-year-old man who was diagnosed with UC at age 22. He first started developing bloating and gastrointestinal pain, with intermittent constipation and loose stools with occasional blood, a year prior, at age 21. A colonoscopy confirmed UC with involvement of the sigmoid and descending colon. He was started on oral and suppository mesalamine, with occasional use of hydrocortisone enema during flares. Symptoms worsened to the point where he was having >12 bloody bowel movements per day and multiple hospitalizations for gastrointestinal symptoms and malnutrition-related bradycardia. During this time, he was also experimenting with various diets, including a specific carbohydrate diet, a low-fermentable oligosaccharides, disaccharides, monosaccharides, and polyols (FODMAP) diet, and a whole-food vegetarian/vegan diet, with dietary trials lasting at least 4 weeks. Then, ~2 years following diagnosis and minimal response to medications and other dietary treatments, he tried a KD. He states, “within a week, my UC symptoms were gone. I had more energy than I’d had in years.” This corresponded with a decrease in fecal calprotectin from previously elevated levels to a low level of 20.4 μg/g. Since that time, he has remained flare-free except for two occasions, both when trying to reintroduce carbohydrates. His KD is centered on animal-based protein sources, although it also includes a large amount of extra virgin olive oil. Similar to patient IA, E manifests the LMHR phenotype with the most recent lipids, LDL-C of 521 mg/dL, HDL-C of 125 mg/dL, and triglycerides of 49 mg/dL. CAC and CCTA reveal no calcified or non-calcified plaque to date.

#### Patient perspective

A ketogenic diet changed my life course. It resurrected my future when I thought I had none. I’ve dabbled with different forms of ketogenic diet, including carnivore, and continue to do so out of personal interest; but anytime I exit ketosis for a prolonged period, my symptoms return. If I eat this way for the rest of my life, that’s fine by me. No starch or sweet is worth not having bloody diarrhea a dozen times per day.

### Case 10

Patient E is a 63-year-old woman with a family history of IBD in her mother and son and a personal medical history significant for psoriasis. She was diagnosed with UC at age 41. She first presented with persistent loose, bloody stools 10–12 times daily for months along with tenesmus, cramping and bloating, and anemia with fatigue. She was initially treated with corticosteroids for flares and methotrexate, which was discontinued for oral mucositis and hair loss. At age 43, she was started on infliximab, which helped put her UC symptoms in remission, except for the return of loose bloody stools when she tried to space her injections. At age 61, she was switched to ustekinumab, under the advice of her rheumatologist in consultation with her gastroenterologist, for the treatment of unmanaged psoriasis. The psoriasis did not improve with ustekinumab. At age 61, E tried a KD for weight loss. She has previously tried dietary patterns focusing on caloric restriction but without any major success. Shortly after starting the diet, which consists of ~90% red meat, eggs, and cheese with some low-carbohydrate vegetables (cauliflower, broccoli, mushrooms, and sauerkraut), she noticed complete resolution of her psoriasis. In the following months, she also lost ~70 lbs, dropping from a BMI of 40 kg/m^2^ to 28.7 kg/m^2^. Noting the significant dermatological response, she gradually spaced her ustekinumab and discontinued the medication 9 months after starting a carnivore-KD, remaining in remission with “100% symptom resolution” to date.

#### Patient perspective

My carnivore-ketogenic diet healed it all! After over 20 years, I’m disease and medication free! I was told that diet could not help me, but I guarantee you it helped. I lived it. I will never buy another candy bar again. And that’s something because I love sweets, but not as much as I love feeling this way.

## Discussion

### Biological plausibility and mechanisms

Of the three features of KD and carnivore diets discussed in the introduction (carbohydrate reduction, dietary elimination, and ketosis), the latter two are of greatest interest in the treatment of IBD.

With respect to therapeutic elements of ketosis, human patients with IBD exhibit reduced levels of ketone and β-hydroxybutyrate (β-HB) in the colonic mucosa, and lower β-HB correlates with higher IBD activity (18). Correspondingly, exogenous β-HB improved colitis by reprogramming macrophages to an anti-inflammatory phenotype via a STAT-6-dependent signaling pathway (18).

With respect to therapeutic elements, elimination diets, reduced intake of processed foods and those that may be “autoimmune triggering”—although a broad and vague description—may provide relief by removing environmental stimuli irritating particularly sensitive gastrointestinal and immune systems. With respect to carnivore diets, specifically, there may also be a role for fiber elimination in IBDs. Recently, Kuffa et al. reported in *Cell Host & Microbe* a mechanism whereby fiber elimination may improve Crohn’s disease (19). For clinical context, fiber-free exclusive enteral nutrition diets are already known to be highly effective at inducing remission (60–85% of cases) in Crohn’s disease; however, such liquid fiber-free diets are considered restrictive and unpalatable, thus limiting their clinical use. In an animal model, Kuffa et al. found that fiber elimination induces the intraluminal migration of the pathobiont *Mucispirillum* through a shift in microbiota hydrogen metabolism in a manner that parallels human patients with Crohn’s disease treated with liquid fiber-free diets. While human carnivory is not mentioned in the Kuffa et al.’s study, it is clearly stated that fiber elimination can be therapeutically effective and that a major obstacle to clinical application is the “poor palatability” of standard fiber-free liquid formulations. The possibility that a palatable, whole-food, low-fiber KD or carnivore diet approach might prove similarly therapeutic is provocative.

### Concerns about cholesterol

This report highlights the therapeutic potential of KD and carnivore diets for IBD. Like any strict diet, these eating patterns are not without potential drawbacks. One of these may be the effects on lipids, with some patients—albeit a minority—exhibiting increases in LDL-C to extremely high levels. While the etiologies of hyperlipidemia on a KD are heterogenous, lower BMI and markers of insulin sensitivity are associated with larger increases as part of a triad of high LDL-C, high HDL-C, and low triglycerides known as the LMHR phenotype (15). Diets deficient in various micronutrients also carry potential risks for chronic diseases; therefore, proper formulation of therapeutic diets is critical and should be conducted in a thoughtful and informed manner prior to commencing the diet, ideally with follow-up to monitor for nutrient deficiencies. For more on this topic and the authors’ thoughts on clinical management, which are beyond the scope of this report, please see our *Journal of Clinical Lipidology* editorial, written with colleague cardiologists and lipidologists: Norwitz et al. (16).

### Suggested future research

As the saying goes, “absence of evidence is not evidence of absence.” To date, there have been no rigorous interventional trials on KD or carnivore diet for IBD, including UC and Crohn’s disease. Nevertheless, with the rising popularity of various forms of carbohydrate-restricted diets, there are accumulating clinical cases of IBD remission upon adopting a KD or carnivore diet. Future research should prioritize testing the efficacy of these diets prospectively. Ideally, we feel a parallel-arm, 8-week randomized controlled feeding trial of an animal-based KD (carnivore diet) versus a whole-food plant-(vegan) would be of interest and yield highly clinically useful data.

## Limitations and strengths

As a case series, the results presented in this article are subject to selection bias: we sought and reported on patients who reported improvement in IBD. Nevertheless, during the recruitment process, we received no cases of patients with IBD responding that a KD or carnivore diet exacerbated their condition, and final inclusion was determined by order of response to solicitation prior to the clinical interview. Nevertheless, selection bias remains a limitation, and this retrospective case series should not be interpreted as advocating that all cases of IBD will improve with a KD or carnivore diet. More study is needed to determine the efficacy of KD and carnivore diets in IBD and factors predicting responsiveness.

Furthermore, given the nature of recruitment, there was also selection bias toward animal-based and fully carnivore KD, as opposed to plant-based KD. We also do not have sufficient data from these cases to make claims as to what diet or patient-specific factors might improve efficacy. From these cases, it seems some patients are less tolerant of plant matter than others, whereas others may be more reliant on ketosis.

Thus, questions remain: what are the relative benefits of ketosis versus processed food elimination? Does fiber elimination have benefits, and—if so—what might be the long-term consequences on IBD and overall health? Are UC and Crohn’s disease differentially responsive to these and other dietary factors, and/or what patient/disease factors might be associated with the relative importance of dietary factors?

Notable strengths of this report include the diversity of case presentations and the fact that all patients had histologically confirmed IBD that went into remission with the commencement of a KD or carnivore diet, with symptoms only returning when patients diverged from their diet.

## Conclusion

These cases highlight the promise of KD and carnivore diets for the treatment of IBD, including UC and Crohn’s disease. Other new data on the anti-inflammatory effects of ketosis in IBD and its effects on the microbiome provide biological plausibility for these clinical reports. More prospective research is urgently needed to explore the potential efficacy of KD and carnivore diets for IBD.

## Data Availability

The datasets presented in this article are not readily available because it contains private patient information. Requests to access the datasets should be directed to the corresponding authors.

## References

[1] Martin-McGill KJ Bresnahan R Levy RG Cooper PN. Ketogenic diets for drug-resistant epilepsy. Cochrane Database Syst Rev. (2020) 2020:CD001903. doi: 10.1002/14651858.CD001903.pub5, PMID: 32588435 PMC7387249

[2] Norwitz NG Hurn M Espi Forcen F. Animal-based ketogenic diet puts severe anorexia nervosa into multi-year remission: a case series. J Metab Health. (2023) 6:8. doi: 10.4102/jir.v6i1.84

[3] Norwitz NG Sethi S Palmer CM. Ketogenic diet as a metabolic treatment for mental illness. Curr Opin Endocrinol Diabetes Obes. (2020) 27:269–74. doi: 10.1097/MED.0000000000000564 32773571

[4] Tomlinson KL Chen YT Junker A Urso A Wong Fok Lung T Ahn D. Ketogenesis promotes tolerance to *Pseudomonas aeruginosa* pulmonary infection. Cell Metab. (2023) 35:1767–1781.e6. doi: 10.1016/j.cmet.2023.09.001, PMID: 37793346 PMC10558090

[5] Cukoski S Lindemann CH Arjune S Todorova P Brecht T Kuhn A. Feasibility and impact of ketogenic dietary interventions in polycystic kidney disease: KETO-ADPKD-a randomized controlled trial. Cell Rep Med. (2023) 4:101283. doi: 10.1016/j.xcrm.2023.101283, PMID: 37935200 PMC10694658

[6] Phillips M Deprez L Mortimer G Murtagh D McCoy S Mylchreest R. Randomized crossover trial of a modified ketogenic diet in Alzheimer’s disease. Alzheimers Res Ther. (2021) 13:51. doi: 10.1186/s13195-021-00783-x, PMID: 33622392 PMC7901512

[7] Ebbeling CB Feldman HA Klein GL Wong JMW Bielak L Steltz SK. Effects of a low carbohydrate diet on energy expenditure during weight loss maintenance: randomized trial. BMJ. (2018) 363:k4583. doi: 10.1136/bmj.k4583, PMID: 30429127 PMC6233655

[8] Ludwig DS. Carbohydrate-insulin model: does the conventional view of obesity reverse cause and effect? Philos Trans R Soc Lond Ser B Biol Sci. (2023) 378:20220211. doi: 10.1098/rstb.2022.0211, PMID: 37661740 PMC10475871

[9] Soto-Mota A Pereira MA Ebbeling CB Aronica L Ludwig DS. Evidence for the carbohydrate-insulin model in a reanalysis of the diet intervention examining the factors interacting with treatment success (DIETFITS) trial. Am J Clin Nutr. (2023) 117:599–606. doi: 10.1016/j.ajcnut.2022.12.014 36811468

[10] Youm YH Nguyen KY Grant RW Goldberg EL Bodogai M Kim D. The ketone metabolite beta-hydroxybutyrate blocks NLRP3 inflammasome-mediated inflammatory disease. Nat Med. (2015) 21:263–9. doi: 10.1038/nm.3804, PMID: 25686106 PMC4352123

[11] Shimazu T Hirschey MD Newman J He W Shirakawa K Le Moan N. Suppression of oxidative stress by beta-hydroxybutyrate, an endogenous histone deacetylase inhibitor. Science. (2013) 339:211–4. doi: 10.1126/science.1227166, PMID: 23223453 PMC3735349

[12] Huang H Zhang D Weng Y Delaney K Tang Z Yan C. The regulatory enzymes and protein substrates for the lysine beta-hydroxybutyrylation pathway. Sci Adv. (2021) 7:eabe2771. doi: 10.1126/sciadv.abe2771, PMID: 33627428 PMC7904266

[13] Lennerz BS Mey JT Henn OH Ludwig DS. Behavioral characteristics and self-reported health status among 2029 adults consuming a "carnivore diet". Curr Dev Nutr. (2021) 5:nzab133. doi: 10.1093/cdn/nzab133, PMID: 34934897 PMC8684475

[14] Budoff M Manubolu VS Kinninger A Norwitz NG Feldman D Wood TR. Carbohydrate restriction-induced elevations in LDL-cholesterol and atherosclerosis: the KETO trial. Metab Clin Exp. (2024) 153:155854. doi: 10.1016/j.metabol.2024.155854 PMC11450898 39372369

[15] Norwitz NG Feldman D Soto-Mota A Kalayjian T DS L. Elevated LDL-cholesterol with a carbohydrate-restricted diet: evidence for a ‘lean mass hyper-responder’ phenotype. Curr Dev Nutr. (2021) 6:nzab144. doi: 10.1093/cdn/nzab144, PMID: 35106434 PMC8796252

[16] Norwitz NG Mindrum MR Giral P Kontush A Soto-Mota A Wood TR. Elevated LDL-cholesterol levels among lean mass hyper-responders on low-carbohydrate ketogenic diets deserve urgent clinical attention and further research. J Clin Lipidol. (2022) 16:765–8. doi: 10.1016/j.jacl.2022.10.010, PMID: 36351849

[17] Soto-Mota A Flores-Huardo Y Norwitz N Feldman D Pereira M Danaei G. Increased LDL cholesterol in adults with normal but not high body weight: a meta-analysis. Am J Clin Nutr. (2024) 119:740–7. doi: 10.1016/j.ajcnut.2024.01.009 38237807

[18] Huang C Wang J Liu H Huang R Yan X Song M. Ketone body beta-hydroxybutyrate ameliorates colitis by promoting M2 macrophage polarization through the STAT6-dependent signaling pathway. BMC Med. (2022) 20:148. doi: 10.1186/s12916-022-02352-x, PMID: 35422042 PMC9011974

[19] Kuffa P Pickard JM Campbell A Yamashita M Schaus SR Martens EC. Fiber-deficient diet inhibits colitis through the regulation of the niche and metabolism of a gut pathobiont. Cell Host Microbe. (2023) 31:2007–2022.e12. doi: 10.1016/j.chom.2023.10.016 37967555 PMC10842462

