# Supplementary material for: Case report: Carnivore–ketogenic diet for the treatment of inflammatory bowel disease: a case series of 10 patients
Source: Front Nutr. 2024 Sep 2;11:1467475. doi: 10.3389/fnut.2024.1467475 (PMC11409203; doi:10.3389/fnut.2024.1467475)
Supplement: Supplementary file 1 [file Table_1.pdf]

Table S1. Analysis of prospective food logs. Data extracted from detailed patient food logs, including food masses and volumes using USDA Food Central database. Data are given as daily average. Pro, protein; Sat, saturated; Unsat, unsaturated.

Patient	Fat, % kCal	Pro, % kCal	Sat:Unsat fatty acid ratio	Carbs, net (g)	Fiber (g)	kCal (total)
1 (TH)	**67%**	30%	**1:2**	21	**17**	2798
2 (AN)	**73%**	25%	**1:1.56**	11	**9**	3076
3 (OS)	**70%**	27%	**1:1.63**	19	**0**	2680
4 (IA)	**71%**	27%	**1:1**	14	**0**	3007
5 (MI)	**68%**	32%	**1:1.63**	~1	**0**	4219
6 (NE)	**67%**	31%	**1:1.08**	19	**0**	3771
7 (VI)	**72%**	27%	**1:1.3**	3	**2**	1848
8 (TA)	**68%**	32%	**1:1.7**	~1	**0**	2730
9 (BL)	**82%**	17%	**1:3**	9	**12**	2738
10 (E)	**72%**	27%	**1:1.5**	5	**23**	1645
